# Serotonin inhibits axonal regeneration of identifiable descending neurons after a complete spinal cord injury in lampreys

**DOI:** 10.1242/dmm.037085

**Published:** 2019-02-20

**Authors:** Daniel Sobrido-Cameán, Diego Robledo, Laura Sánchez, María Celina Rodicio, Antón Barreiro-Iglesias

**Affiliations:** 1 Department of Functional Biology, CIBUS, Faculty of Biology, Universidade de Santiago de Compostela, 15782 Santiago de Compostela, Spain; 2 The Roslin Institute and Royal (Dick) School of Veterinary Studies, The University of Edinburgh, Midlothian EH25 9RG, UK; 3 Department of Genetics, University of Santiago de Compostela, Campus de Lugo, 27002 Lugo, Spain

**Keywords:** 5-HT, Serotonin receptor 1A, cAMP, Descending neurons, Fish, Axon regeneration, CNS

## Abstract

Classical neurotransmitters are mainly known for their roles as neuromodulators, but they also play important roles in the control of developmental and regenerative processes. Here, we used the lamprey model of spinal cord injury to study the effect of serotonin in axon regeneration at the level of individually identifiable descending neurons. Pharmacological and genetic manipulations after a complete spinal cord injury showed that endogenous serotonin inhibits axonal regeneration in identifiable descending neurons through the activation of serotonin 1A receptors and a subsequent decrease in cyclic adenosine monophosphate (cAMP) levels. RNA sequencing revealed that changes in the expression of genes that control axonal guidance could be a key factor determining the serotonin effects during regeneration. This study provides new targets of interest for research in non-regenerating mammalian models of traumatic central nervous system injuries and extends the known roles of serotonin signalling during neuronal regeneration.

This article has an associated First Person interview with the first author of the paper.

## Abstract (precis)

**Summary:** Pharmacological and genetic manipulations show that endogenous serotonin inhibits axonal regeneration of individually identifiable descending neurons of lampreys after a complete spinal cord injury.

## INTRODUCTION

In contrast to mammals, including humans, lampreys recover locomotion spontaneously following a complete spinal cord injury (SCI) (see Barreiro-Iglesias, 2012, 2015; Rodicio and Barreiro-Iglesias, 2012; Sobrido-Cameán and Barreiro-Iglesias, 2018). In lampreys, the process of recovery from SCI involves a positive astroglial response (Fernández-López et al., 2014), the production of new neurons in the spinal cord (Zhang et al., 2014), and the regeneration of ascending and descending axons through the injury site (Yin and Selzer, 1983; Davis and McClellan, 1994; Jacobs et al., 1997; Cornide-Petronio et al., 2011). This regeneration is specific in that axons grow selectively in their normal directions (Yin and Selzer, 1983; Yin et al., 1984; Mackler et al., 1986). Moreover, regenerated axons of descending brain neurons are able to re-establish synaptic connections with their appropriate targets below the site of injury (Mackler and Selzer, 1987; Oliphint et al., 2010). However, in lampreys, not all descending neurons of the brain are able to regenerate their axons following a complete spinal cord transection, even when normal-appearing locomotor function is observed several weeks after the injury (Davis and McClellan, 1994; Jacobs et al., 1997). Approximately 50% of all descending brainstem neurons are able to regenerate their axon below the site of injury after a complete SCI (Cohen et al., 1986; Oliphint et al., 2010). Among brain descending neurons, the brainstem of lampreys contains several giant individually identifiable descending neurons that vary greatly in their regenerative ability, even when their axons run in similar paths in a spinal cord that is permissive for axonal regrowth (Jacobs et al., 1997). Some identifiable neurons, such as the Mauthner or I1 neurons, regenerate their axon less than 10% of the times after a complete spinal cord transection, while other identifiable neurons, such as the I3 or B6 neurons, are able to regenerate their axon 60% of the times after a complete SCI (Jacobs et al., 1997). This suggests that intrinsic factors that are present in some neurons, but not in others, might limit their regenerative ability. Lampreys offer a convenient vertebrate model in which to study the inhibition or promotion of axonal regeneration after SCI in the same *in vivo* preparation.

One of the molecules known to be a key intrinsic regulator of axonal regeneration is the second messenger cyclic adenosine monophosphate (cAMP) (see Hannila and Filbin, 2008; Hao et al., 2016). Several reports in mammals and fishes have shown that cAMP promotes axon regeneration following SCI (Neumann et al., 2002; Qiu et al., 2002; Bhatt et al., 2004; Nikulina et al., 2004; Pearse et al., 2004). Subsequent studies have also shown that cAMP promotes axon regeneration in descending neurons of lampreys after SCI (Jin et al., 2009; Lau et al., 2013; Pale et al., 2013). The challenge now is to define the signals that control cAMP levels in descending neurons after axotomy and during regeneration.

Several neurotransmitters modulate intracellular cAMP levels by activating metabotropic G-protein-coupled receptors, which include serotonin (5-HT), glutamate, γ-aminobutyric acid (GABA) or dopamine receptors. So, neurotransmitters acting through these receptors are potential regulators of intracellular cAMP levels following a traumatic injury to the central nervous system (CNS). Among them, 5-HT appears as a good candidate to regulate axon regeneration following nervous system injuries (see Sobrido-Cameán et al., 2018). 5-HT receptors are divided into seven families, with families 1, 2 and 4-7 being G-protein-coupled metabotropic receptors, and family 3 of the 5-HT receptors being ligand-gated ion channels. Families 1 and 5 of the 5-HT receptors are known to decrease intracellular levels of cAMP, whereas families 4, 6 and 7 increase intracellular levels of cAMP. A few *in vitro* studies have shown that 5-HT inhibits axon regrowth in invertebrate (Murrain et al., 1990; Koert et al., 2001) and vertebrate (Lima et al., 1994, 1996) species. In contrast, a recent study in *Caenorhabditis elegans* showed that 5-HT promotes axon regeneration after axotomy (Alam et al., 2016). However, no study has as yet looked at the role of 5-HT in axon regeneration in an *in vivo* vertebrate model of traumatic CNS injury. The existence of rich 5-HT innervation in the vicinity of descending neurons of the lamprey brainstem (Antri et al., 2006; Abalo et al., 2007; Antri et al., 2008; Barreiro-Iglesias et al., 2008), the expression of 5-HT1A receptors in identifiable descending neurons of lampreys (Cornide-Petronio et al., 2013), electrophysiological data showing that descending neurons of lampreys are modulated by 5-HT (Antri et al., 2008) and data showing an increase in synaptic contacts on descending neurons following SCI in lampreys (Lau et al., 2011) prompted us to study the possible role of 5-HT in axon regeneration following SCI in lampreys. Here, we present gain- and loss-of-function data, using pharmacological and genetic treatments, showing that endogenous 5-HT inhibits axon regeneration in identifiable descending neurons of lampreys following a complete SCI by activating 5-HT1A receptors. We also performed an RNA sequencing study, which revealed that changes in the expression of genes that control axonal guidance could be a key factor in determining the effects of 5-HT during regeneration. This provides a new target of interest for SCI research in non-regenerating mammalian models.

## RESULTS

### A 5-HT treatment inhibits axon regeneration in identifiable descending neurons after a complete SCI

**Fig. 1.**
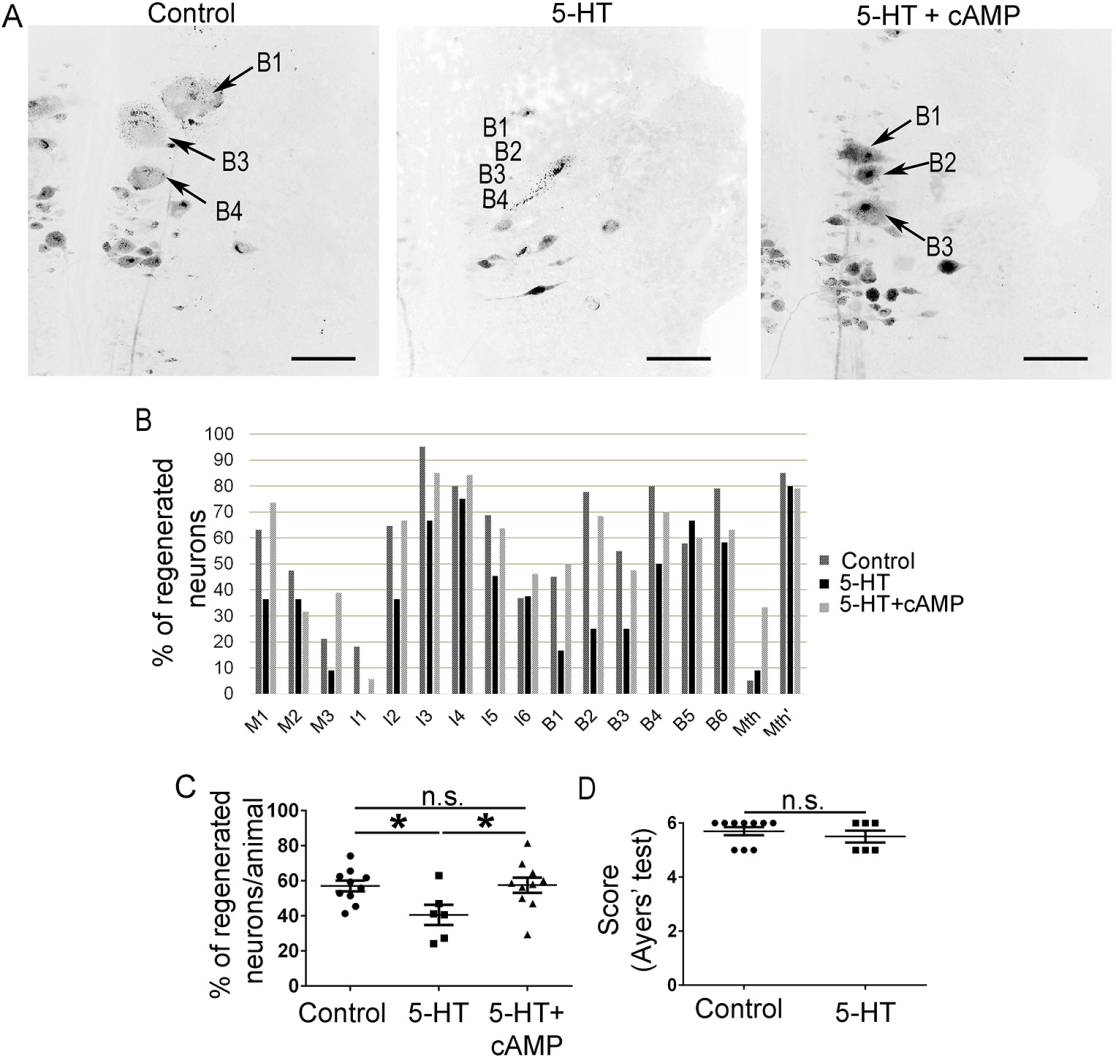
**A 5-HT treatment inhibits axonal regeneration of identifiable descending neurons and an additional cAMP treatment rescues the inhibitory effect of 5-HT.** (A) Photomicrographs of whole-mounted brains showing regenerated identifiable neurons, as identified by retrograde labelling, in control, 5-HT-treated, and 5-HT- and cAMP-treated animals. Note the decreased number of labelled (regenerated) identifiable neurons in 5-HT-treated animals. Arrows indicate identifiable descending neurons that regenerated in control or 5-HT- and cAMP-treated animals, but not in 5-HT-treated animals. Rostral is up and the midline to the left in all photomicrographs. Scale bars: 100 µm. (B) Graph showing the percentage of regenerated neurons (with respect to the total number of analyzed neurons) for each identifiable descending neuron in control, 5-HT-treated, and 5-HT- and cAMP-treated animals. (C) Graph showing significant changes (*P≤0.05) in the percentage of identifiable regenerated neurons per animal after the 5-HT or 5-HT and cAMP treatments (control: 57.06±3.08%; 5-HT: 40.56±5.74%; 5-HT+cAMP: 57.51±4.39%). (D) Graph showing non-significant (n.s.) differences in recovery of swimming behaviour (Ayers’ test) in control and 5-HT-treated animals (Ayers' test score: control: 5.70±0.15; 5-HT treated: 5.50±0.22).

To reveal the effect of 5-HT in the regeneration of identifiable descending neurons, larval sea lampreys were treated with the 5-HT analogue 5-HT-hydrochloride for a month following a complete spinal cord transection. At 11 weeks post-lesion (wpl), the 5-HT treatment significantly inhibited axon regeneration of identifiable descending neurons of the sea lamprey (Student's *t*-test, *P*=0.0145; Fig. 1A-C). Importantly, behavioural analyses revealed that the 5-HT treatment did not cause a general toxic effect since locomotor recovery was not affected by the 5-HT treatment (Fig. 1D).

### cAMP treatment can reverse the inhibitory effect of 5-HT on axonal regeneration

Previous studies have shown that a single dose of cAMP applied at the time of transection promotes axon regeneration of identifiable descending neurons following a complete spinal cord transection in larval sea lampreys (Jin et al., 2009; Lau et al., 2013; Pale et al., 2013). Here, and to reveal a possible involvement of this second messenger in the inhibitory effect of 5-HT in axonal regeneration, we carried out a rescue experiment in which animals treated with 5-HT as above were also treated with dibutyryl-cAMP (db-cAMP). The db-cAMP treatment was able to significantly rescue the inhibitory effect of 5-HT, with these animals reaching levels of axonal regeneration of identifiable descending neurons similar to control vehicle-treated animals (ANOVA, *P*=0.0288; Fig. 1A-C).

### Serotonergic system and identifiable descending neurons after SCI

Previous studies showed the presence of rich serotonergic innervation in the vicinity of descending neurons in the brainstem of lampreys and that the activity of these neurons is modulated by 5-HT (see Introduction). Here, we carried out 5-HT immunofluorescence experiments combined with tracer labelling from the site of a complete SCI. We also observed the presence of rich serotonergic innervation in close proximity to the injured identifiable descending neurons (see an example in Fig. S1). So, identifiable descending neurons can receive endogenous 5-HT signalling after a complete SCI.

The 5-HT1A receptor is an ancient G-protein-coupled receptor that is known to decrease intracellular cAMP levels through G_i_/G_o_ when activated by 5-HT. In a previous study, we showed that this receptor is expressed in identifiable descending neurons of larval sea lampreys (Cornide-Petronio et al., 2013). This, together with the results of the db-cAMP treatments (see previous section) prompted us to study whether there was a correlation between the expression of the 5-HT1A receptor in identifiable descending neurons and their known regenerative ability following a complete spinal cord transection. We carried out *in situ* hybridization analyses in horizontal sections of the sea lamprey brain to look at the expression of the 5-HT1A receptor in individually identifiable neurons. This, as opposed to whole-mounts, impedes the clear identification of all identifiable descending neurons in all sections; therefore, in these analyses only the M1, M2, M3, I1 and I3 neurons were included.

**Fig. 2.**
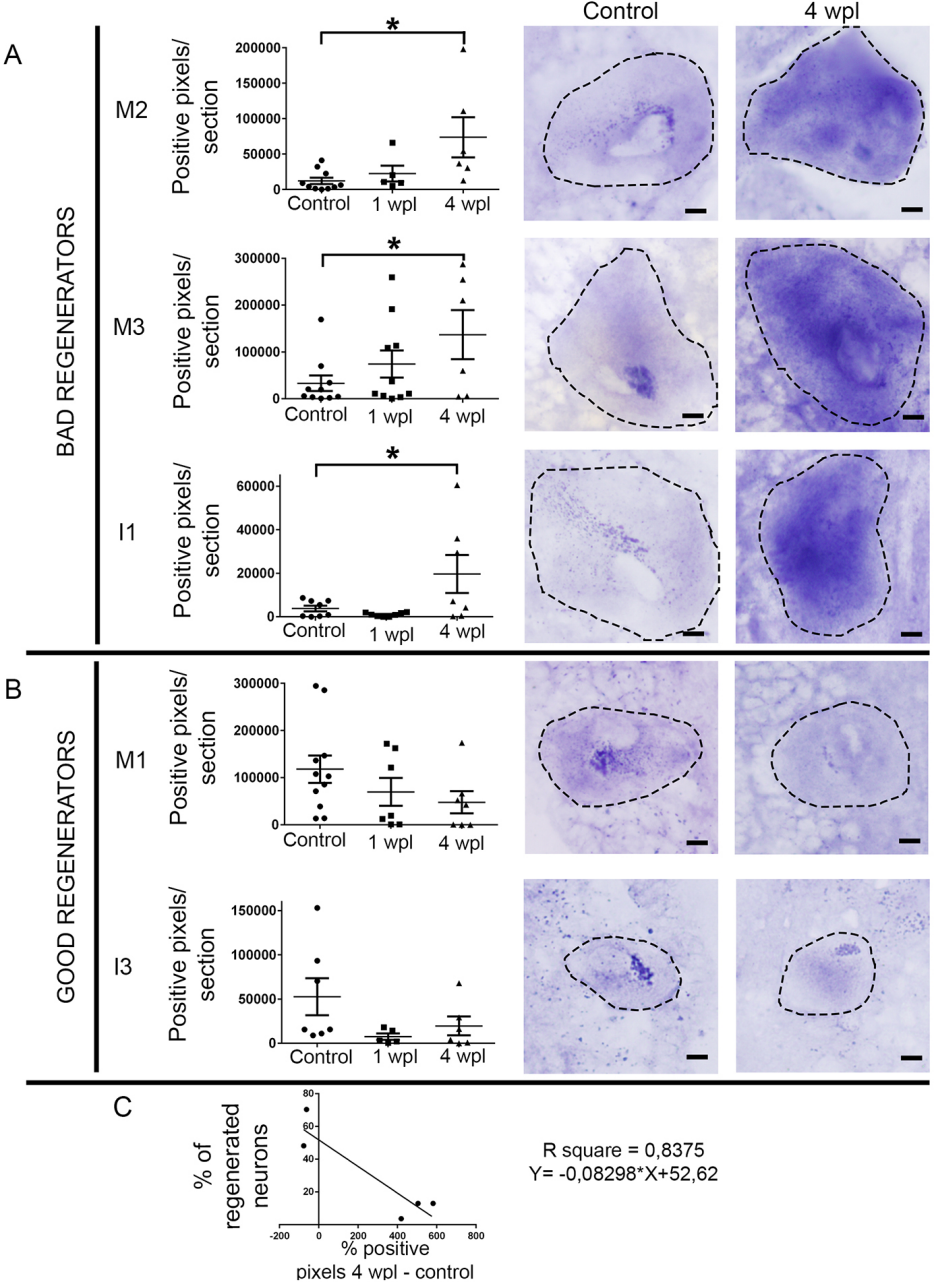
**Changes in the expression of the 5-HT1A receptor in identifiable descending neurons after a complete SCI.** (A) Graphs and photomicrographs showing significant changes (*P≤0.05) in the number of 5-HT1A-positive *in situ* pixels per section of the soma of bad-regenerator identifiable descending neurons. The mean±s.e.m. values are provided in Table 2. (B) Graphs and photomicrographs showing changes in the number of 5-HT1A-positive *in situ* pixels per section of the soma of good-regenerator identifiable descending neurons. The mean±s.e.m. values are provided in Table 2. Examples of photomicrographs of horizontal sections of control and injured identifiable descending neurons are shown to the right of the graphs in A and B. The somas of identifiable neurons are outlined in the photomicrographs. The location of these neurons in the sea lamprey brain can be observed in Fig. S3. Scale bars: 20 µm. (C) Linear regression analysis showed an inverse relation between the percentage of change in *5-HT1A* transcript expression at 4 wpl and regeneration probability (from Jacobs et al., 1997) of each cell (95% confidence intervals for slope=−0.08298±0.0211; *S*_y/x_=13.23).

Our *in situ* hybridization experiments revealed that, in ‘bad regenerator’ neurons (neurons that regenerate their axon less than 30% of the times after a complete spinal cord transection), there is a significant increase in the expression of the 5-HT1A receptor 4 weeks after a complete SCI (M2 neuron: Kruskal–Wallis, *P*=0.0105; M3 neuron: ANOVA, *P*=0.0478; I1 neuron: ANOVA, *P*=0.0238; Fig. 2A; Table 1), whereas, in ‘good regenerator’ neurons (M1 and I3 neurons), the expression of the receptor decreases (non-significantly) in the first weeks following a complete spinal cord transection (Fig. 2B; Table 1). Statistical analyses revealed a significant correlation between the percentage of increase/decrease in the expression of the receptor at 4 wpl and the long-term regenerative ability of identifiable neurons (Pearson's test, *P*=0.0293, Fig. 2C). These data, together with the 5-HT immunofluorescence results, suggest that the presence and activity of the 5-HT1A receptor in descending neurons might inhibit axonal regeneration after a complete SCI.

**Table 1.**
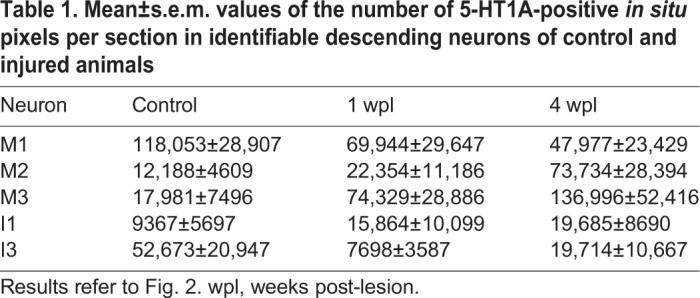
**Mean±s.e.m. values of the number of 5-HT1A-positive *in situ* pixels per section in identifiable descending neurons of control and injured animals**

**Table 2.**
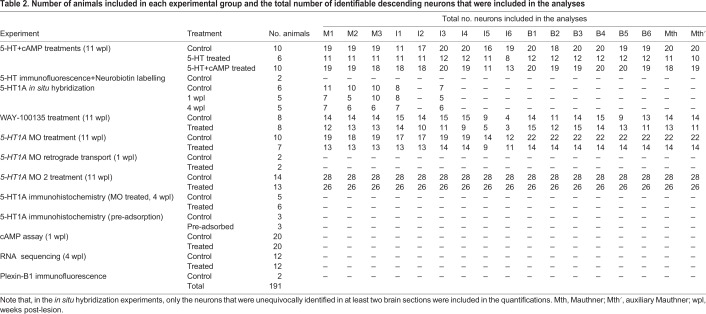
**Number of animals included in each experimental group and the total number of identifiable descending neurons that were included in the analyses**

### Endogenous 5-HT inhibits axon regeneration through the 5-HT1A receptor following SCI

**Fig. 3.**
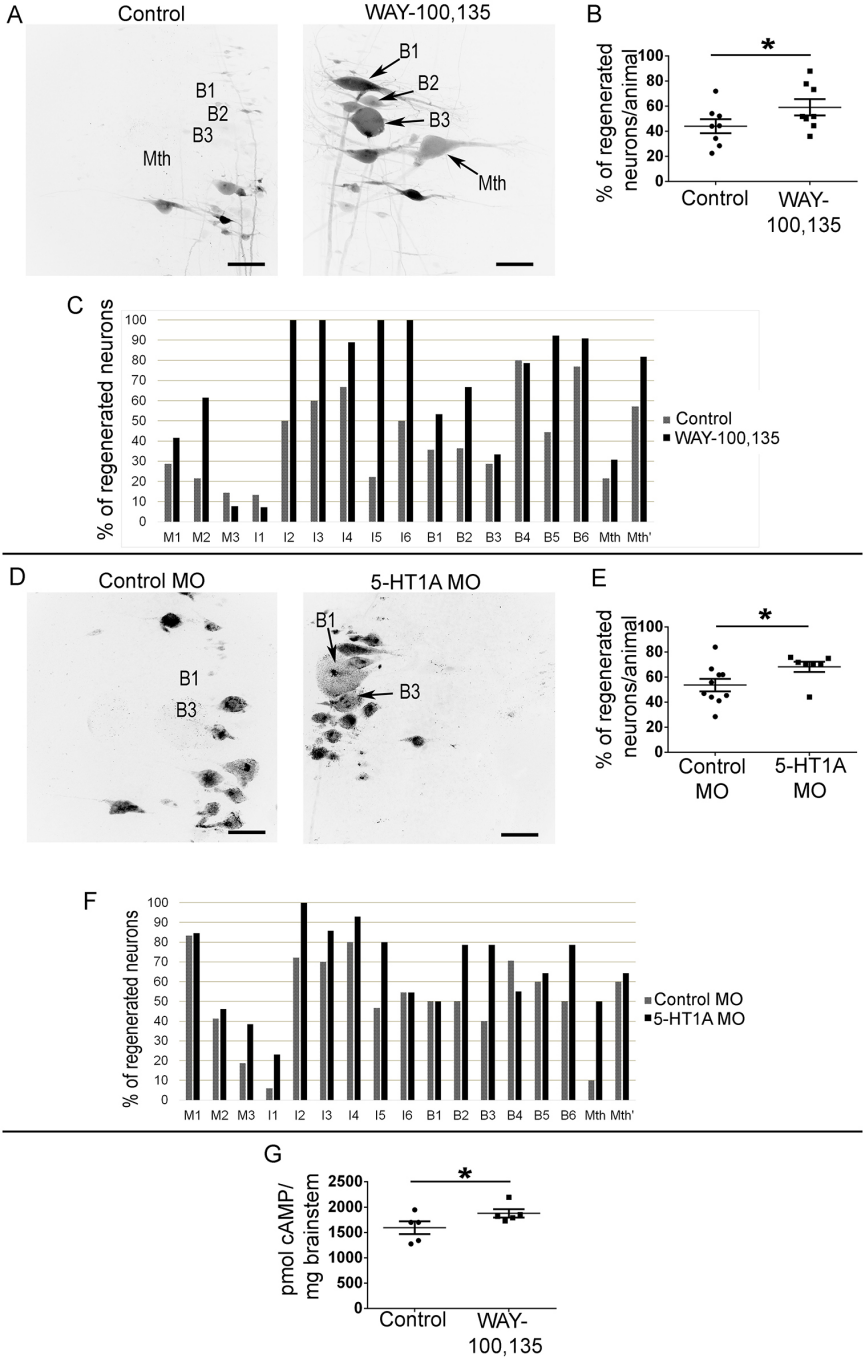
**WAY-100135 or *5-HT1A* MO treatments promote axonal regeneration of identifiable descending neurons.** (A) Photomicrographs of whole-mounted brains showing regenerated identifiable neurons, as identified by retrograde labelling, in control and WAY-100135-treated animals. Note the increased number of labelled (regenerated) identifiable neurons in WAY-100135-treated animals. (B) Graph showing significant changes (*P≤0.05) in the percentage of regenerated neurons per animal after the WAY-100135 treatment (control: 44.05±5.58%; WAY-100135: 59.16±6.44%). (C) Graph showing the percentage of regenerated neurons (with respect to the total number of analyzed neurons) for each identifiable cell in control and WAY-100135-treated animals. (D) Photomicrographs of whole-mounted brains showing regenerated identifiable neurons, as identified by retrograde labelling, in control- and *5-HT1A*-MO-treated animals. Note the increased number of labelled (regenerated) identifiable neurons in *5-HT1A* MO-treated animals. (E) Graph showing significant changes (*P≤0.05) in the percentage of regenerated neurons per animal after the *5-HT1A* MO treatment (control: 53.69± 4.97%; 5-HT1A MO: 68.29±4.12%). (F) Graph showing the percentage of regenerated neurons (with respect to the total number of analyzed neurons) for each identifiable cell in control- and *5-HT1A*-MO-treated animals. (G) Graph showing significant changes (*P≤0.05) in cAMP concentration per milligram of brainstem in control and WAY-100135-treated animals (control: 1.594±125.4 pmol of cAMP/mg of brainstem; WAY-100135: 1.878± 81.74 pmol of cAMP/mg of brainstem). (A,D) Arrows indicate descending neurons that regenerated in WAY-100135 or *5-HT1A*-MO-treated animals but not in control animals. Rostral is up in all photomicrographs. The midline is to the right in photomicrographs of control animals and to the left in photomicrographs of treated animals. Scale bars: 100 µm.

Based on the immunofluorescence and *in situ* hybridization results, we decided to study whether endogenous 5-HT inhibits axon regeneration of identifiable descending neurons by activating the 5-HT1A receptors. For this, we treated animals for 4 weeks after a complete spinal cord transection with the 5-HT1A receptor antagonist WAY-100135. The WAY-100135 treatment significantly promoted axon regeneration 11 wpl in identifiable neurons compared to control vehicle-treated animals (Student's *t*-test, *P*=0.049, Fig. 3A-C). This indicates that endogenous 5-HT inhibits axon regeneration following a complete SCI by activating 5-HT1A receptors. The WAY-100135 treatment did not significantly change the behavioural recovery of the animals (Ayers' test: control: 5.875±0.125; WAY-100135: 5.875±0.081; Mann–Whitney *U*-test, *P*>0.9999; not shown), although this was expected since control animals already reach the highest degrees of recovery (5 to 6).

To confirm that the inhibitory effect of endogenous 5-HT is due to the activation of 5-HT1A receptors expressed in identifiable descending neurons, we decided to specifically knock down the expression of the receptor in these neurons by using morpholinos targeted against the translation initiation site of the 5-HT1A receptor (Fig. S2A). The morpholinos were applied at the time of transection on the rostral stump of the spinal cord. Fluorescent labelling of the morpholinos confirmed that these were retrogradely transported and reached the soma of descending neurons during the first wpl (Fig. S2B). Immunohistochemical analyses revealed that, at 4 wpl, the application of the morpholino targeting the translation initiation site significantly reduced the expression of the 5-HT1A receptor in reticulospinal neurons of the sea lamprey brainstem as compared to the standard control morpholino (Student's *t*-test, *P*=0.001; Fig. S2C,D). More importantly, and as expected from the antagonist treatments, the application of this morpholino significantly promoted axon regeneration of identifiable neurons following a complete spinal cord transection as compared to the animals that received the standard control morpholino (Mann–Whitney *U*-test, *P*=0.0177, Fig. 3D-F). A second morpholino targeting the 5′ untranslated region of the *5-HT1a* mRNA (non-overlapping with the first morpholino) was used as a control of specificity (Fig. S2A). The application of this second morpholino also significantly promoted axon regeneration of identifiable neurons (Student's *t*-test, *P*=0.0342; Fig. S2E). Both the antagonist and morpholino treatments indicate that endogenous 5-HT inhibits axon regeneration in identifiable neurons after SCI by activating 5-HT1A receptors expressed in these neurons.

### The WAY-100135 treatment increases cAMP levels in the brainstem

To study whether changes in cAMP levels might be behind the beneficial effects of the 5-HT1A receptor antagonist treatment, another set of animals was treated with WAY-100135 for 1 week. A cAMP detection assay of the whole brainstem revealed that the antagonist treatment, which also promoted axonal regeneration (see previous section), significantly increased cAMP levels in the brainstem (Mann–Whitney *U*-test, *P*=0.0397; Fig. 3G). This result indicates that the inhibition of axon regeneration caused by the activation of 5-HT1A receptors by endogenous 5-HT might be caused by a decrease in intracellular levels of cAMP.

### RNA sequencing following a WAY-100135 treatment reveals new signalling pathways possibly involved in axonal regeneration in lampreys

Our gain- and loss-of-function experiments revealed that endogenous 5-HT inhibits axon regeneration following SCI in the sea lamprey through the activation of 5-HT1A receptors and that this effect might be caused by a decrease in intracellular cAMP levels. To reveal new genes that might be involved in the intrinsic control of axon regeneration and whose expression is modulated by the activity of 5-HT1A receptors, we decided to repeat the WAY-100135 treatment and carry out an RNA sequencing study in the sea lamprey brainstem at 4 wpl.

**Fig. 4.**
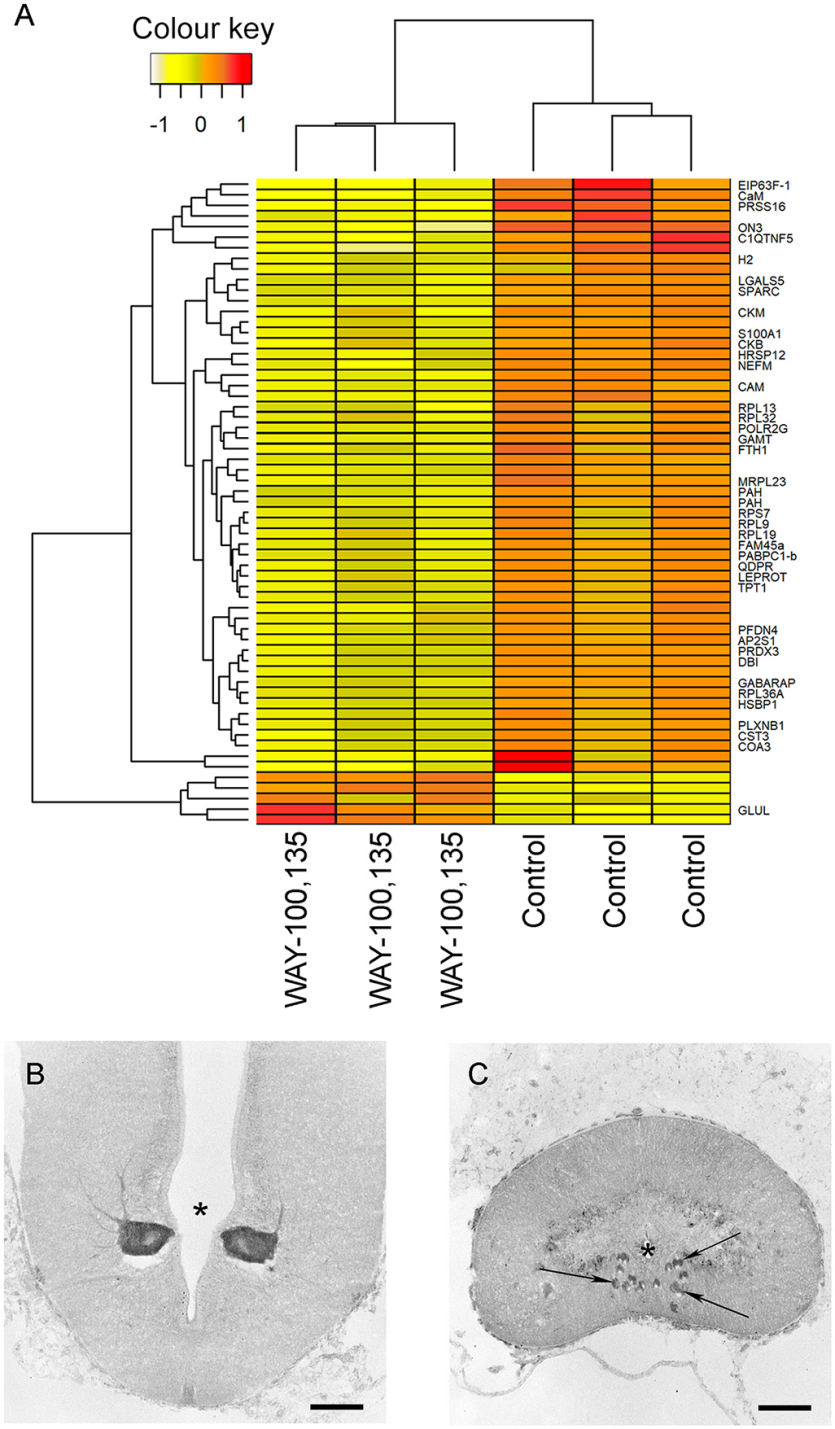
**The WAY-100135 treatment causes changes in gene expression.** (A) Heatmap showing the expression of 61 differentially expressed genes between samples treated with WAY-100135 and control samples in RNA sequencing. Gene expression is represented as regularized log-transformed read counts scaled by gene. Samples (*x*-axis) and genes (*y*-axis) were hierarchically clustered according to their gene expression Euclidian distances using complete linkage clustering. (B) Photomicrograph of a transverse section of the sea lamprey brain showing the presence of intense Plexin-B1 immunoreactivity in the M2 neuron. (C) Photomicrograph of a transverse section of the sea lamprey spinal cord showing the presence of Plexin-B1 immunoreactivity in descending axons (arrows). The asterisks indicate the ventricle (B) and central canal (C). Dorsal is to the top. The plane of section of these photomicrographs can be observed in Fig. S3. Scale bars: 100 µm.

A total of 61 differentially expressed genes were detected between WAY-100135-treated samples and control vehicle-treated animals (Fig. 4A, Table S1). Most of these genes were found to be downregulated after WAY-100135 treatment (5 upregulated genes, 56 downregulated). Among these downregulated genes in response to WAY-100135 is Plexin-B1 (*PLXNB1*, logFC=−0.42), which plays a role in axon guidance and works as a transmembrane receptor for semaphorins (Driessens et al., 2002). Only one of the five significantly upregulated genes was annotated, Glutamine synthetase (*GLUL*, logFC=1.05). This gene is primarily found in astrocytes and protects neurons against excitotoxicity (Suarez et al., 2002). Pathway analysis using Reactome (Fabregat et al., 2018) revealed 29 significantly enriched pathways (FDR *P*-value<0.05; Table S2). Among these, the most interesting ones are ‘Axon guidance’, ‘Signalling by ROBO receptors’ and ‘Regulation of expression of SLITs and ROBOs’, which are represented only by downregulated genes.

Since this RNA sequencing analysis was performed using the whole brainstem, which contains both descending and non-descending neurons, we decided to confirm the expression of one of the ‘Axon guidance’ genes, Plexin-B1, in descending neurons. Immunofluorescence experiments revealed the presence of intense Plexin-B1 immunoreactivity in the soma (Fig. 4B) and axons (Fig. 4C) of identifiable descending neurons, which confirms that these neurons can be a direct target of 5-HT signalling after SCI.

## DISCUSSION

We have provided gain- and loss-of-function evidence, using pharmacological and genetic treatments, showing that endogenous 5-HT inhibits axon regeneration after SCI in lampreys by activating 5-HT1A receptors expressed in descending neurons. Our results also suggest that differential changes in 5-HT1A receptor expression in descending neurons could be one of the factors that explain the different regenerative abilities of individual descending neurons.

Only a few studies have previously looked at the possible role of 5-HT in neurite/axon regeneration, and these have been carried out only in invertebrate and *in vitro* models (see Sobrido-Cameán et al., 2018). Koert and co-workers (2001) showed that, in the snail *Lymnaea stagnalis*, auto-released 5-HT inhibited neurite outgrowth in cultured serotonergic cerebral giant cells. An *ex vivo* study in the pond snail (*Helisoma trivolis*) showed that the spontaneous regeneration of specific neurons was inhibited by 5-HT treatments (Murrain et al., 1990). 5-HT or 5-HT-reuptake-inhibitor treatments also inhibited neurite outgrowth from goldfish retinal explants with a previous crush to the optic nerve (Lima et al., 1994, 1996). So, our results confirm these previous *in vitro* studies and show that endogenous 5-HT inhibits axon regeneration after a traumatic injury *in vivo* and in a vertebrate species. The only exception to these results comes from a recent study showing that, in the nematode *C. elegans*, 5-HT promotes axon regeneration (Alam et al., 2016). In these animals, non-serotonergic neurons temporarily express tryptophan hydroxylase (the rate-limiting enzyme in 5-HT synthesis) in response to axotomy, promoting their regeneration (Alam et al., 2016). We should consider that 5-HT can signal through a variety of 5-HT receptors that activate different secondary pathways. In our study, the db-cAMP co-treatment rescued the inhibitory effects of the 5-HT treatment (see above). This effect of db-cAMP could be additive and independent of 5-HT, but our results using a 5-HT1A receptor antagonist indicate that, in lampreys, the negative effect of endogenous 5-HT on the regeneration of descending neurons is caused by the activation of 5-HT1A receptors and a subsequent reduction in cAMP levels. Previous *in vitro* studies in goldfish also showed that the activation of 5-HT1A receptors inhibits neurite outgrowth (Lima et al., 1994; Schmeer et al., 2001). Accordingly, 5-HT1A agonist treatment reduced neurite outgrowth from retinal explants of goldfish with a prior crush of the optic nerve (Lima et al., 1994; Schmeer et al., 2001). Interestingly, in *C. elegans*, the opposite effect of 5-HT was mediated by the activation of 5-HT7 receptors, since mutations of this receptor caused defects in axon regeneration after axotomy (Alam et al., 2016). Notably, Alam and co-workers (2016) showed that the positive effect of the activation of this receptor was caused (at least partially) by the stimulation of adenylate cyclase to produce cAMP. Taken together, present and previous results indicate that 5-HT is an important regulator of axon regeneration by modulating cAMP levels, and that the effects of 5-HT will depend on the preferential use of different types of 5-HT receptors in different neurons.

Our RNA sequencing study also revealed changes in gene expression in the brainstem associated with 5-HT1A receptor signalling after a complete SCI. Of interest, a treatment with a 5-HT1A receptor antagonist (which increases cAMP levels and promotes axon regeneration) reduced the expression of some genes associated with the regulation of axonal guidance. Specifically, Plexin-B1 was one of the most significantly downregulated genes, together with the pathways ‘Axon guidance’, ‘Signalling by ROBO receptors’ and ‘Regulation of expression of SLITs and ROBOs’. Interestingly, previous work in lampreys has suggested that the expression/activity of some receptors that participate in axonal guidance inhibits axonal regeneration after SCI in identifiable neurons (e.g. UNC5: Shifman and Selzer, 2000; Barreiro-Iglesias et al., 2012; Chen et al., 2017). Also, a previous study already suggested a possible involvement of semaphorin/plexin signalling in recovery after SCI in lampreys based on the occurrence of changes in the expression of semaphorins in the spinal cord after the injury (Shifman and Selzer, 2007). Future studies should functionally test the possible involvement of Slits and semaphorins in the inhibition of axonal regeneration in descending neurons after SCI in lampreys.

Present and previous work in regenerating species shows that 5-HT plays different roles in the process of regeneration after SCI. In zebrafish, endogenous 5-HT promotes motor neuron production in the spinal cord after a complete SCI by enhancing the proliferation of motor neuron progenitor cells (Barreiro-Iglesias et al., 2015). In turtles, 5-HT inhibits the emergence of serotonergic interneurons after SCI by inhibiting a change in neurotransmitter phenotype of non-serotonergic neurons (Fabbiani et al., 2018). On the other hand, the production of new-born serotonergic neurons in the spinal cord of zebrafish after SCI is not affected by endogenous 5-HT (Barreiro-Iglesias et al., 2015). Here, our results show that, in regenerating vertebrates, endogenous 5-HT also controls axon regeneration of descending neurons after SCI. These studies suggest that 5-HT could be a target of interest in non-regenerating mammalian models of SCI, since it can modulate several aspects of the regenerative process. Even more importantly, the effects of 5-HT revealed in these studies should be considered by those authors performing pharmacological treatments with serotonergic drugs to modulate spinal cord circuits aiming to promote locomotor recovery after SCI (see Becker and Parker, 2015), mainly because these treatments could be affecting the regeneration of new neurons or the re-growth of axotomized axons as shown here.

## MATERIALS AND METHODS

### Animals

All experiments involving animals were approved by the Bioethics Committee at the University of Santiago de Compostela and the Consellería do Medio Rural e do Mar of the Xunta de Galicia (license reference JLPV/IId; Spain), and were performed in accordance with European Union and Spanish guidelines on animal care and experimentation. During the experimental procedures, special effort was taken to minimize animal suffering and to reduce the use of animals. Animals were deeply anaesthetized with 0.1% MS-222 (Sigma, St Louis, MO) in lamprey Ringer solution before all experimental procedures and euthanized by decapitation at the end of the experiments.

Mature and developmentally stable larval sea lampreys, *Petromyzon marinus* L. (*n*=191; between 90 and 120 mm in body length, 5-7 years of age), were used in the study. Larval lampreys were collected from the river Ulla (Galicia, north-western Spain), with permission from the Xunta de Galicia, and maintained in aerated freshwater aquaria at 15°C with a bed of river sediment until their use in experimental procedures. Larval lampreys were randomly distributed between the different experimental groups.

### SCI surgical procedures

Larval sea lampreys were assigned to the following experimental groups: control animals without a complete spinal cord transection or animals with a complete spinal cord transection that were analyzed 1 wpl, 4 wpl or 11 wpl. Within some of the 4 wpl and 11 wpl groups, the animals were assigned to either a vehicle-treated control group or to a treatment group. Table 2 summarizes the number of animals assigned to each experimental group. Each experiment was carried out in at least two different batches of animals. Complete spinal cord transections were performed as previously described (Barreiro-Iglesias et al., 2014). Briefly, the spinal cord was exposed from the dorsal midline at the level of the fifth gill by making a longitudinal incision with a scalpel (#11 blade). A complete spinal cord transection was performed with Castroviejo scissors and the spinal cord cut ends were visualized under the stereomicroscope. Then, the animals were kept on ice for 1 h to allow the wound to air dry. After this hour, the animals were returned to freshwater tanks and each transected animal was examined 24 h after surgery to confirm that there was no movement caudal to the lesion site. Then, the animals were allowed to recover in freshwater tanks at 19.5°C and in the dark.

### Drug treatments

The following drugs were used to treat the animals following the complete spinal cord transection: 5-HT-hydrochloride (a 5-HT analogue that crosses the blood-brain barrier; AlfaAesar; Cat#: B21263; MW: 212.68 g/mol), WAY-100135 (a 5-HT1A receptor antagonist that crosses the blood-brain barrier; Sigma; Cat#: W1895; MW: 468.46 g/mol) and db-cAMP (Sigma; Cat#: D0260; MW: 491.37 g/mol). 5-HT-hydrochloride was applied at a concentration of 500 µM in the water in which the animals were left after the surgical procedures. Controls were left in freshwater only. Animals were treated with 5-HT-hydrochloride for 4 wpl, changing the water and the drug twice per week. WAY-100135 was dissolved in lamprey Ringer's solution (137 mM NaCl, 2.9 mM KCl, 2.1 mM CaCl_2_, 2 mM HEPES; pH 7.4) and injected intraperitoneally at a concentration of 1 mM (volume of 25 µl per injection). Vehicle injections served as a control. Animals were treated with WAY-100135 for 1 or 4 wpl, receiving 1 intraperitoneal injection per week. Dd-cAMP was also dissolved in lamprey Ringer's solution at a concentration of 100 mM, soaked in a small piece of Gelfoam (Pfizer; New York, NY) and placed on top of the site of injury at the time of transection as previously described by other authors (Jin et al., 2009; Lau et al., 2013). Gelfoam soaked in the same volume of lamprey Ringer's solution served as a control.

We assumed that these drugs also crossed the blood-brain barrier in lampreys as in mammals, since the blood-brain barrier of lampreys is similar to that of higher vertebrates (Bundgaard, 1982; Bundgaard and van Deurs, 1982). 5-HT was applied at the same concentration previously used by other authors (Becker and Parker, 2015). Becker and Parker (2015) already reported that this concentration of 5-HT affects the swimming behaviour of lesioned and unlesioned animals, indicating that this application route allows access to the CNS. We also observed changes in the swimming behaviour of unlesioned animals in pilot experiments using WAY-100135 at this concentration (not shown). For db-cAMP application, we followed the protocol used by other authors of studies on lampreys, in which this treatment promoted axonal regeneration after SCI (Jin et al., 2009; Lau et al., 2013).

### 5-HT immunofluorescence combined with retrograde tract-tracing

Immunofluorescence experiments were carried out to confirm the presence of serotonergic innervation in the vicinity of injured identifiable neurons of 1 wpl animals. The retrograde tracer Neurobiotin (NB, 322.8 Da molecular mass; Vector Labs, Burlingame, CA) was applied on the rostral end of the transected spinal cord with the aid of a minute pin (#000). The animals were allowed to recover at 19°C with appropriate ventilation conditions for 1 week to allow transport of the tracer from the application point to the soma of descending neurons.

For immunohistochemistry, the brains of larvae were fixed by immersion in 4% paraformaldehyde (PFA) in 0.05 M Tris-buffered saline pH 7.4 (TBS) for 4 h at 4°C. The samples were then rinsed in TBS, cryoprotected with 30% sucrose in TBS, embedded in Tissue Tek (Sakura), frozen in liquid-nitrogen-cooled isopentane, and cut serially on a cryostat (14 µm thickness) in transverse planes. Sections were mounted on Superfrost^®^ Plus glass slides (Menzel). Then, the sections were incubated with a rabbit polyclonal anti-5-HT antibody (dilution 1:2500; Immunostar, Still Water, MN; Cat#: 20080; lot 431001; RRID: AB_572263; immunogen: 5-HT-formaldehyde-BSA conjugate) at room temperature overnight. The primary antibody was diluted in TBS containing 15% normal goat serum and 0.2% Triton X-100 as detergent. The sections were rinsed in TBS and incubated for 1 h at room temperature with a Cy3-conjugated goat anti-rabbit antibody (1:200; Millipore; Burlington, MA). Then, the sections were incubated at room temperature with Avidin D-FITC conjugated (Vector; Cat#: A-2001; 1:1000) diluted in TBS containing 0.3% Triton X-100 for 4 h to reveal the presence of Neurobiotin. Slides were rinsed in TBS and distilled water, and mounted with Mowiol.

The specificity of the anti-5-HT antibody was tested by the supplier, who reported no detectable cross-reactivity with tryptamine, 5-methoxytryptamine, L-tryptophan, 5-hydroxytryptophan, dopamine, norepinephrine (noradrenaline) or adrenaline. The anti-5-HT antibody was tested by western blot of sea lamprey brain extracts in our laboratory (Villar-Cerviño et al., 2006). No protein band was detected in these blots. Moreover, immunostaining of sections was completely abolished after pre-adsorption of the anti-5-HT antibody with 5-HT–BSA conjugates (Abalo et al., 2007).

### *In situ* hybridization

For 5-HT1A receptor *in situ* hybridization, the head of the animals was fixed by immersion in 4% PFA in 0.05 M Tris-buffered saline (TBS; pH 7.4) for 12 h. Then, the brains were dissected out, washed and embedded in Neg 50™ (Microm International GmbH, Walldorf, Germany), frozen in liquid-nitrogen-cooled isopentane, sectioned on a cryostat in the horizontal plane (14 μm thick) and mounted on Superfrost Plus glass slides (Menzel, Braunschweig, Germany). *In situ* hybridization with a specific riboprobe for the 5-HT1A receptor (GenBank accession number KU314442.1) was conducted as previously described (Cornide-Petronio et al., 2013, 2014). Briefly, brain sections were incubated with the sea lamprey 5-HT1A receptor DIG-labelled probe at 70°C and treated with RNAse A (Invitrogen, Massachusetts, USA) in the post-hybridization washes. Then, the sections were incubated with a sheep anti-DIG antibody conjugated to alkaline phosphatase (1:2000; Roche, Mannhein, Germany) overnight. Staining was conducted in BM Purple (Roche) at 37°C. *In situ* hybridization experiments were performed in parallel with animals from the different experimental groups (control, 1 wpl and 4 wpl) and the colorimetric reaction was stopped simultaneously for all sections from the different groups of animals.

### Morpholino treatments

The spinal cord was transected at the level of the fifth gill (see ‘SCI surgical procedures’ section), and 1 µl of the morpholinos (0.25 mM in Milli-Q-water) were applied on the rostral stump of the spinal cord. The morpholinos were designed by GeneTools, LLC (Philomath, OR) and included two fluorescein-conjugated active translation-blocking 5-HT1A receptor morpholinos (first 5-HT1A receptor morpholino: 5′-CTGTGATGTTGTGAGCTTCCATCG-3′; second 5-HT1A receptor morpholino: 5′-CGCTCGTCTTTGTGTGGA-3′) generated against the translation initiation region of the sea lamprey 5-HT1A receptor sequence (Fig. S2A), and the fluorescein-conjugated GeneTools standard control morpholino (5′-CCTCTTACCTCAGTTACAATTTATA-3′). During recovery, the morpholinos are retrogradely transported to the cell soma of descending neurons where they can knock down the expression of the target mRNA (Fig. S2B; Zhang et al., 2015; Fogerson et al., 2016; Chen et al., 2017; Hu et al., 2017; Romaus-Sanjurjo et al., 2018). The GeneTools standard control morpholino has already been used in previous studies using morpholino treatments after SCI in lampreys (Zhang et al., 2015; Hu et al., 2017). Animals were allowed to recover for 1, 4 or 11 wpl.

Immunofluorescence experiments were carried out as above (for 5-HT immunofluorescence) to confirm that the active 5-HT1A receptor morpholino decreases the expression of the sea lamprey receptor. In these experiments, the sections were incubated with a rabbit polyclonal anti-5-HT1A receptor antibody (dilution 1:200; Abcam; Cambridge, UK; Cat#: ab85615; lot GR318082-1; RRID: AB_10696528; immunogen: synthetic peptide corresponding to rat 5-HT1A receptor amino acids 100-200 conjugated to keyhole limpet haemocyanin) at room temperature overnight. 0.2% Tween was used as a detergent in these experiments. Amino acids 100-200 of the rat receptor show an 87% correspondence with the same region of the sea lamprey 5-HT1A receptor. Moreover, immunostaining of sea lamprey sections was abolished after pre-adsorption of the anti-5-HT1A receptor antibody with the synthetic peptide (Fig. S2F).

### Behavioural analyses

The behavioural recovery of the animals treated with 5-HT-hydrochloride or WAY-100135 was analyzed at 11 wpl based on the study of Ayers (1989) and following the protocol of Hoffman and Parker (2011) before tracer application. This qualitative assessment of locomotor function was made from video recordings of 5 min (camera: Panasonic Full-HD HC-V110). The animals were placed in a plastic aquarium (36×23×10.5 cm) and swimming activity was initiated by lightly pinching the tail of the animal using a pair of forceps. Locomotor recovery of the animals was categorized in a scale of 1-6 (Ayers, 1989; Hoffman and Parker, 2011). Animals in stage 5 or 6 correspond to animals in which regeneration of axons through the site of injury has occurred based on activity evoked by stimulation across the lesion site in the isolated spinal cord (Hoffman and Parker, 2011). Two blinded experimenters independently evaluated each 11 wpl animal. Based on both analyses, a mean value of locomotor recovery was obtained for each animal.

### Retrograde labelling of regenerated descending neurons

At 11 wpl, a second complete spinal cord transection was performed 5 mm below the site of the original transection and the retrograde tracer Neurobiotin (Vector) was applied in the rostral end of the transected spinal cord with the aid of a minute pin (#000). The animals were allowed to recover at 19°C with appropriate ventilation conditions for 1 week to allow transport of the tracer from the application point to the neuronal soma of descending neurons (the M1, M2, M3, I1, I2, I3, I4, I5, I6, B1, B2, B3, B4, B5, B6, Mauthner (Mth) and auxiliary Mauthner (Mth′) neurons were analyzed; Fig. S3). Since the original SCI also was a complete spinal cord transection, only neurons whose axons regenerated at least 5 mm below the site of injury were labelled by the tracer. Brains of these larvae were dissected out, the posterior and cerebrotectal commissures of the brain were cut along the dorsal midline, and the alar plates were deflected laterally and pinned flat to a small strip of Sylgard (Dow Corning Co., USA) and fixed with 4% PFA in TBS for 4 h at 4°C. After washes in TBS, the brains were incubated at 4°C with Avidin D-FITC conjugated (Vector; Cat#: A-2001; 1:500) diluted in TBS containing 0.3% Triton X-100 for 2 days to reveal the presence of Neurobiotin. Brains were rinsed in TBS and distilled water and mounted with Mowiol.

### Assay for the quantification of cAMP concentration

cAMP concentration in the brainstem of animals treated with WAY-100135 was analyzed 1 wpl using a quantitative cAMP competitive ELISA kit (Invitrogen, Waltham, MA, USA; Cat#: EMSCAMPL) following the manufacturer's instructions. This assay measures cAMP levels in a sample based on the competition of the sample's cAMP with alkaline phosphatase-conjugated cAMP for an anti-cAMP antibody. The anti-cAMP anitbody is bound to an anti-rabbit IgC-pre-coated 96-well plate. The brains of larvae were dissected out 1 h after the second WAY-100135 or control intraperitoneal injections and immediately homogenized in 0.1 M HCl on ice.

### RNA sequencing

Animals treated during 4 wpl with WAY-100135 and control vehicle-treated animals were processed for an RNA sequencing analysis of the whole brainstem. The brainstems of larvae were dissected out and immediately put in RNA*later^®^* (Ambion Inc.). RNA extraction was performed using the RNeasy mini kit (Qiagen) with DNase treatment following the manufacturer's instructions. RNA quality and quantity were evaluated in a Bioanalyzer (Bonsai Technologies) and in a NanoDrop^®^ ND-1000 spectrophotometer (NanoDrop^®^ Technologies Inc.), respectively. Three WAY-100135 and three control samples (each sample containing 4 brainstems) were barcoded and prepared for sequencing by the Wellcome Trust Centre for Human Genetics (Oxford, UK) using standard protocols. Sequencing was conducted on an Illumina HiSeq 2000 as 100 bp paired-end reads. Raw sequencing data have been deposited in NCBI's Short Read Archive (SRA) under BioProject accession PRJNA472778. The quality of the sequencing output was assessed using FastQC v.0.11.5 (http://www.bioinformatics.babraham.ac.uk/projects/fastqc/). Quality filtering and removal of residual adaptor sequences was conducted on read pairs using Trimmomatic v.0.35 (Bolger et al., 2014). Specifically, residual Illumina-specific adaptors were clipped from the reads, leading and trailing bases with a Phred score less than 20 were removed and the read trimmed if a sliding window average Phred score over four bases was less than 20. Only reads where both pairs had a length greater than 36 bp post-filtering were retained. A *de novo* transcriptome was assembled using Trinity v.2.4.0 (Grabherr et al., 2011) with default settings. Gene expression was estimated using Kallisto v.0.43.1 (Bray et al., 2016) and statistical analyses related to differential expression were performed using R v.3.4.3 (http://www.R-project.org/). Gene count data were used to estimate differential gene expression using the Bioconductor package DESeq2 v.3.4 (Love et al., 2014). The Benjamini-Hochberg false discovery rate (FDR) was applied, and transcripts with corrected *P*-values <0.05 were considered differentially expressed genes. Heatmaps were drawn using the R package ‘gplots’ v.3.01.1 (Gregory et al., 2016). Pathway analyses were performed using Reactome (Fabregat et al., 2018), converting all non-human gene identifiers to their human equivalents.

Immunofluorescence experiments were carried out as above (for 5-HT) to confirm Plexin-B1 expression in descending neurons of the sea lamprey. In these experiments, the transverse sections of the brain and spinal cord were incubated with a rabbit polyclonal anti-Plexin-B1 antibody (dilution 1:100; Developmental Studies Hybridoma Bank; Iowa, USA; Cat#: PCRP-PLXNB1-3A7; RRID: AB_2618961; immunogen: human Plexin-B1) at room temperature overnight. The alignment of the partial sea lamprey Plexin-B1 protein sequence and the human plexin-B1 protein sequence shows 41% amino acid identity and 60% positive amino acid positions.

### Imaging and quantifications

The percentage of neurons with regenerated axons (labelled by the Neurobiotin tracer) with respect to the total number of analyzed neurons (see Table 1) was calculated for each type of identifiable descending neuron using an Olympus microscope. The percentage of neurons with regenerated axons with respect to the total number of analyzed neurons in each animal was also calculated and these data were used for statistical analyses. The experimenter was blinded during quantifications. For the figures, images were taken with a spectral confocal microscope (model TCS-SP2; Leica).

An Olympus photomicroscope (AX-70; Provis) with a 20× Apochromatic 0.75 lens and equipped with a colour digital camera (Olympus DP70, Tokyo, Japan) was used to acquire images of brain sections from the *in situ* hybridization experiments. Images were always acquired with the same microscope and software settings. The quantification of the level of 5-HT1A-receptor-positive signal in identifiable descending neurons was performed as previously described (Romaus-Sanjurjo et al., 2018). First, we established the intensity rank of a positive colorimetric *in situ* signal. For this, we analyzed ten random images from different descending neurons of control and lesioned animals. The ‘histogram’ function in ImageJ shows the number of pixels in each image in a range of intensity from 0 to 255. With these images, we compared the intensity values in regions with clear *in situ* signal and the intensity values in regions without an *in situ* signal. Based on this, we established a value of 179 as the lower limit to consider the colorimetric *in situ* signal as positive. Then, the number of pixels of a positive *in situ* signal was quantified for each section of each identified descending neuron. In horizontal brain sections, the identification of some of the specific descending cells becomes more difficult than in whole-mounts. Thus, only the cells that were unequivocally identified in at least two different sections were included in the quantifications (the M1, M2, M3, I1 and I3 neurons; see Fig. S3). Then, we calculated the average number of positive pixels per section for each individual neuron (see Table 1) and these data were used for statistical analyses. The experimenter was blinded during quantifications.

For the quantification of changes in 5-HT1A receptor immunoreactivity after morpholino application, three 14-µm transverse sections of the medial reticular nucleus of the rhombencephalon were analyzed in each animal. One out of every two consecutive sections in the next six sections caudally to the last section where the Mth neuron was observed were analyzed in each animal. The mean fluorescence intensity of each section was calculated using ImageJ and then the mean fluorescence intensity per section in each animal was used for the statistical analyses.

After quantifications, contrast and brightness were minimally adjusted with Adobe Photoshop CS6 (Adobe Systems, San José, CA, USA). Figure plates and lettering were generated using Adobe Photoshop CS6 (Adobe Systems). Schematic drawing was generated using CorelDraw Graphics Suite 2017.

### Statistical analyses

Statistical analysis was carried out using Prism 6 (GraphPad software, La Jolla, CA). Data were presented as means±s.e.m. Normality of the data was determined by the Kolmogorov–Smirnov test or the D'Agostino–Pearson omnibus test. The data from the db-cAMP treatments and the *in situ* hybridization data that were normally distributed were analyzed by a one-way ANOVA. *Post hoc* Dunnett's multiple-comparison tests were used to compare pairs of data. *In situ* hybridization data that were not normally distributed were analyzed by a Kruskal–Wallis test and *post hoc* Dunn's multiple comparisons test. A Student's *t*-test was used to determine significant differences between conditions in the qPCR analysis. The results of control versus treatment groups were analyzed by a Student's *t*-test (normally distributed data) or Mann–Whitney *U*-test (non-normally distributed data). The significance level was set at 0.05. In the figures, significance values were represented by a different number of asterisks in the graphs: *P-value between 0.01 and 0.05, **P-value between 0.001 and 0.01. Exact *P*-values are given in the text.

## Supplementary Material

Supplementary information
