# Supplementary material for: Serotonin inhibits axonal regeneration of identifiable descending neurons after a complete spinal cord injury in lampreys
Source: Dis Model Mech. 2019 Feb 20;12(2):dmm037085. doi: 10.1242/dmm.037085 (PMC6398502; doi:10.1242/dmm.037085)
Supplement: Supplementary information [file dmm-12-037085-s1.pdf]

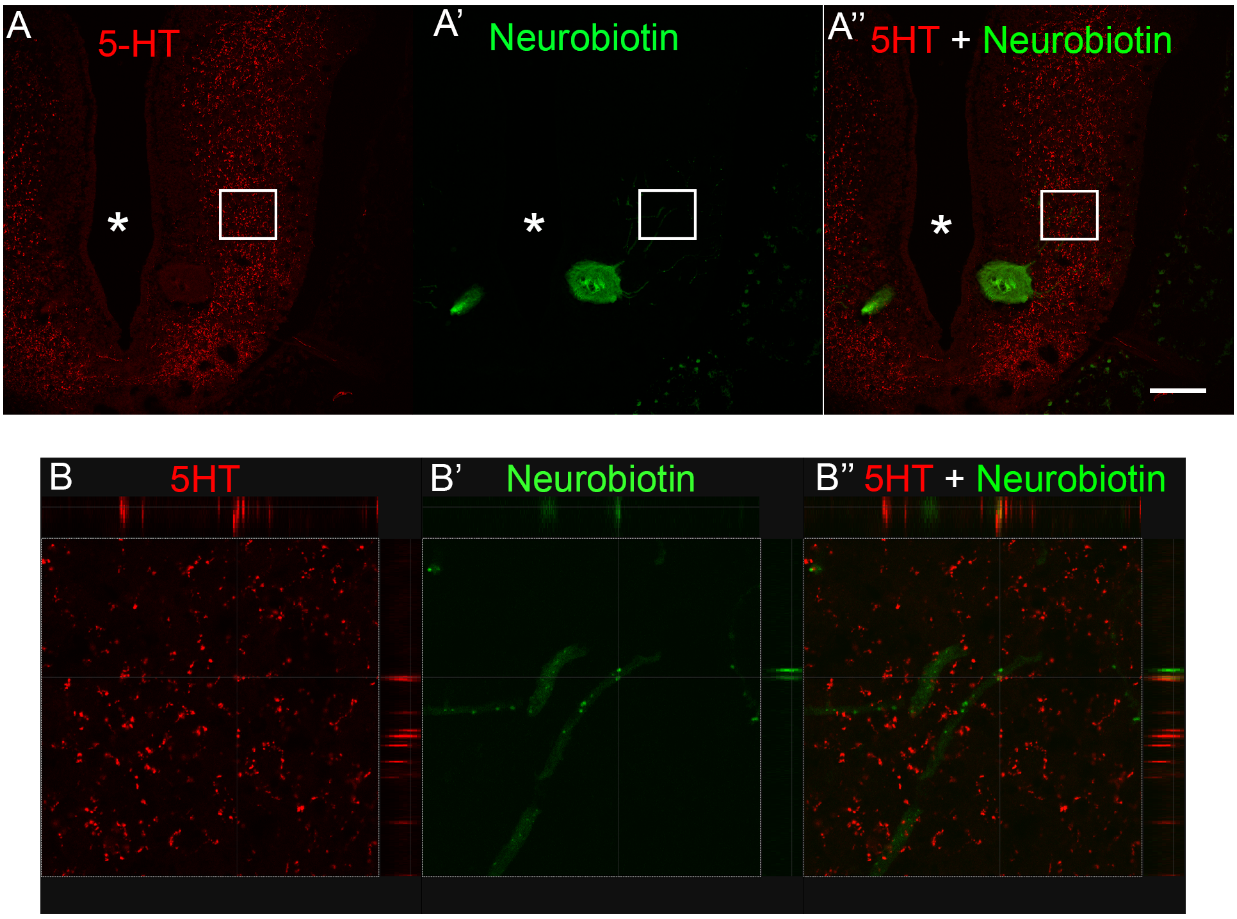

Figure S1. 5-HT innervation of identifiable descending neurons after SCI. A-A'': Photomicrographs of a transverse section of the sea lamprey midbrain showing the presence of rich serotonergic innervation (in red) in the vicinity of the M3 neuron (labelled with Neurobiotin in green). **B-B'':** Orthogonal reconstruction of a detail of the region indicated with a square in **A-A''** showing a serotonergic fibre in very close proximity (see overlapping of both channels in yellow) to a dendrite of the M3 neuron. The asterisks indicate the ventricle. Dorsal is to the top. The plane of section can be observed in Fig. S3. Scale bar: 100 µm.

Disease Models & Mechanisms • Supplementary information

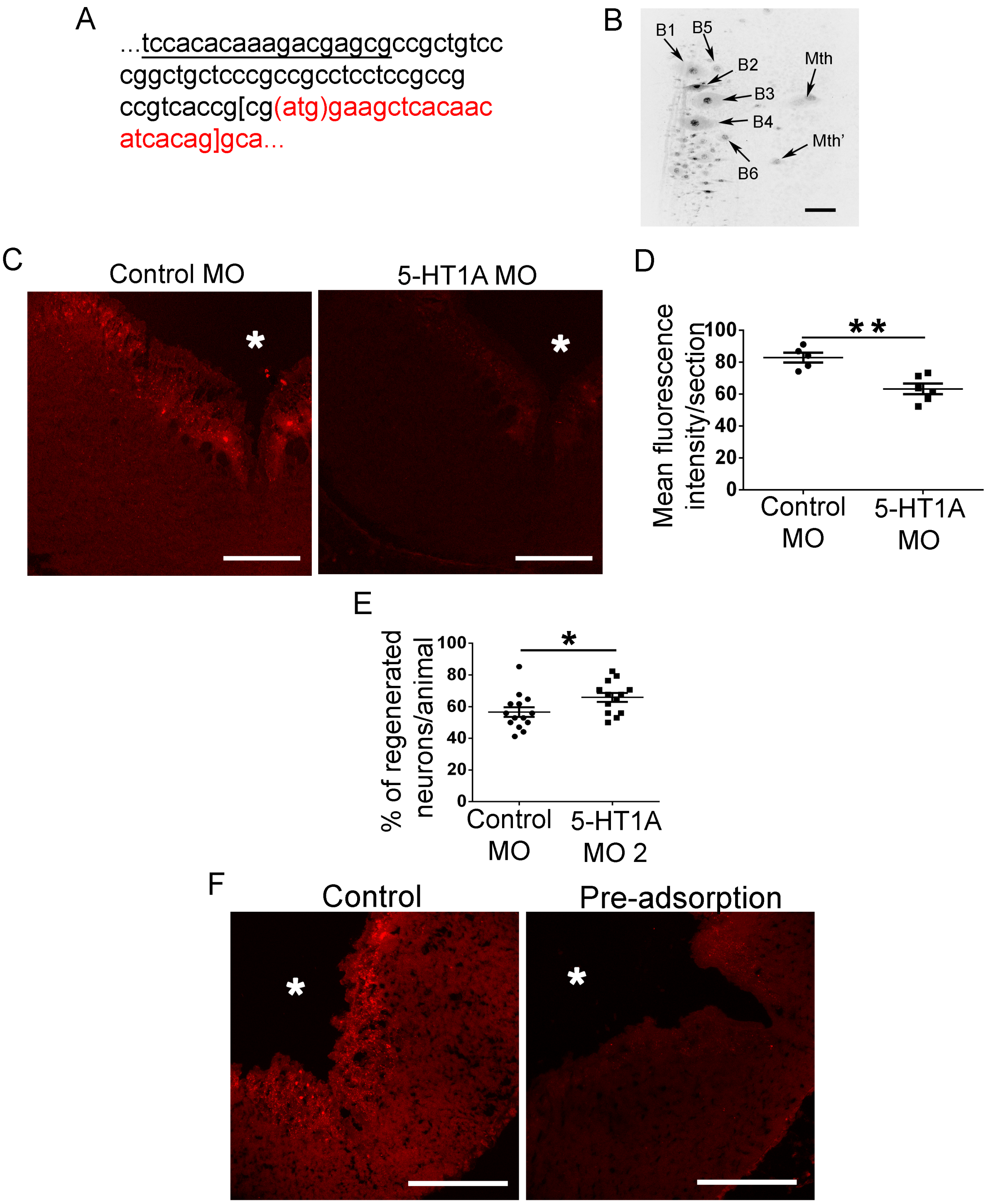

Disease Models & Mechanisms • Supplementary information

Figure S2. 5-HT1A receptor morpholinos. A: Partial sequence of the sea lamprey 5-HT1a receptor with exon sequence in red and 5' untranslated sequence in black. The target sequence of the first 5-HT1A MO is between square brackets (see Fig. 3D-F). The target sequence of the second 5-HT1A MO is underlined. The ATG initiation codon is indicated between parenthesis. **B:** Photomicrograph of a whole-mounted brain showing the presence of the first 5-HT1A MO in identifiable neurons 1 week after the MO application at the site of SCI. **C:** Photomicrographs of transverse sections of the brainstem showing 5-HT1A immunoreactivity in control and 5-HT1A MO treated animals. Note the decreased immunoreactivity in reticulospinal neurons of 5-HT1A MO treated animals. The asterisks indicate the ventricle. Dorsal is to the top in all photomicrographs. The plane of section of these photomicrographs can be observed in Fig. S3. **D:** Graph showing significant changes (asterisks) in the mean fluorescence intensity of 5-HT1A immunoreactivity per section in reticulospinal nuclei of the brainstem after the 5-HT1A MO treatment. **E:** Graph showing significant changes (asterisk) in the percentage of regenerated neurons per animal after the treatment with the second 5-HT1A MO (control: 56.51 ± 3.02 %; 5-HT1A MO 2: 65.84 ± 2.83 %). **F:** Photomicrographs of transverse sections of the brainstem showing 5-HT1A immunoreactivity in control and in anti-5-HT1A antibody pre-adsorption experiments. Note the lack of immunoreactivity in reticulospinal neurons after antibody pre-adsorption. The asterisks indicate the ventricle. Dorsal is to the top in all photomicrographs. The plane of section of these photomicrographs can be observed in Fig. S3. Scale bars: 100 µm.

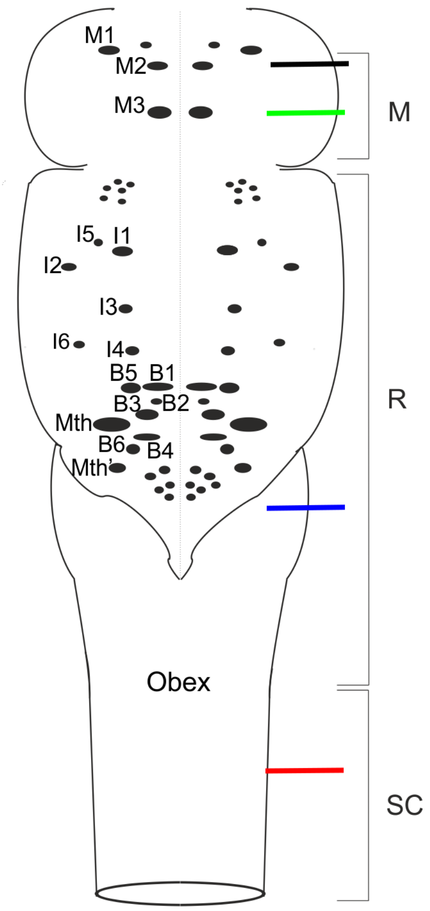

Figure S3. Schematic drawing of a dorsal view of the sea lamprey brainstem showing the location of identifiable descending neurons (modified from Sobrido-Cameán and Barreiro-Iglesias, 2018). The approximate plane of section of Fig. 4B is shown in black. The approximate plane of section of Fig. S1 is shown in green. The approximate plane of section of Figs. S2C and E is shown in blue. The approximate plane of section of Fig. 4C is shown in red. Abbreviations: M: mesencephalon; R: rhombencephalon; SC: spinal cord. Rostral is up.

Supplementary Tables

Table S1. Differentially expressed genes between control and WAY-100,135 treated samples.

List of genes showing significant differential expression (FDR p-value < 0.05) between control samples and samples treated with WAY-100,135. Gene expression level (average normalized count values), log2 fold change (positive values represent up-regulation in response to WAY-100,135 treatment), FDR corrected p-value, Uniprot ID of the most significant blast hit, blast alignment length, blast alignment identity percentage, blast alignment E value, protein name of the most significant blast hit, gene symbol of the most significant blast hit and species of the most significant blast hit are shown for every differentially expressed gene.

Click here to Download Table S1

Table S2. Results of the Reactome pathway analysis.

List of Reactome pathways showing significant enrichment (FDR p-value < 0.05) for the list of differentially expressed genes between control and WAY-100,135 treated samples. Pathway name, total genes assigned to the pathway, total genes present in the pathway and FDR corrected p-value are shown for every enriched Reactome pathway.

Click here to Download Table S2

Disease Models & Mechanisms • Supplementary information
